# Staff perceptions of care navigators in medication for opioid use disorder treatment settings

**DOI:** 10.1186/s13011-026-00743-4

**Published:** 2026-06-27

**Authors:** Michael Goetz, Shaquita Andrews-Higgins, Olivia A. Davis, Sandra Back-Haddix, Laura Fanucchi, Michelle R. Lofwall, Sharon L. Walsh, Hannah K. Knudsen

**Affiliations:** 1 Substance Use Research Priority Area, University of Kentucky, 845 Angliana Ave, Lexington, KY 40508 USA; 2 Present Address: Interdisciplinary Human Development Institute, University of Kentucky, 617 Scenic Drive, Harrodsburg, KY 40330 USA; 3 College of Medicine-Northern Kentucky Campus, University of Kentucky, 100 Grant Dr, Highland Heights, KY 41099 USA; 4 College of Medicine and Center on Drug and Alcohol Research, University of Kentucky, 845 Angliana Avenue, Lexington, KY 40508 USA; 5 Department of Behavioral Science and Center on Drug & Alcohol Research, University of Kentucky, 845 Angliana Avenue, Lexington, KY 40508 USA; 6 Department of Internal Medicine, University of Kentucky, 300 Rose St., Suite C300, Lexington, KY 40536 USA

**Keywords:** Opioid use disorder, Patient navigation, Opioid maintenance treatment, Implementation science, Medication for opioid use disorder, Buprenorphine, Qualitative research, Care navigator

## Abstract

**Background:**

Care navigators are increasingly incorporated into medication for opioid use disorder (MOUD) treatment programs to address barriers to care and support patient engagement and retention. Prior research has largely focused on patient outcomes or program effectiveness, with limited attention to how frontline staff experience the integration of care navigators into routine treatment workflows. This study aimed to examine staff perceptions of care navigator roles and identify organizational and policy-relevant factors influencing their implementation.

**Methods:**

We conducted a qualitative study of staff perceptions of care navigators embedded in medication for opioid use disorder treatment centers participating in the HEALing Communities Study in Kentucky, USA. Semi-structured interviews were conducted with clinical and administrative staff working in participating treatment centers. Interviews explored experiences with care navigator integration, perceived impacts on patient engagement and team functioning, and implementation challenges. Data were analyzed using thematic analysis guided by the Reach, Effectiveness, Adoption, Implementation, and Maintenance framework and the Practical, Robust Implementation and Sustainability Model.

**Results:**

Staff described care navigators as filling critical service gaps by helping patients address practical challenges that commonly disrupt treatment engagement, including transportation barriers, housing instability, and navigating health and social services. These efforts were viewed as supporting sustained engagement in treatment with medication for opioid use disorder. Participants also reported improved team morale and reduced burnout following care navigator integration. However, staff identified persistent implementation challenges, including unclear role boundaries, scheduling constraints, and misalignment between organizational cultures. Clear differentiation between care navigators, targeted case managers, and peer support specialists was consistently identified as necessary to support effective collaboration.

**Conclusions:**

Findings suggest that care navigators may enhance engagement and workforce capacity in medication for opioid use disorder treatment settings, particularly when focused on addressing practical barriers to care. At the same time, variability in role definition and organizational fit limited effectiveness in some settings. These results highlight the importance of clear role delineation, stable funding, and implementation planning when integrating non-clinical navigation roles. Future research should incorporate patient perspectives and examine how policy and organizational contexts shape implementation and outcomes.

**Trial registration:**

ClinicalTrials.gov Identifier: NCT04111939 (registered March 26, 2019).

**Supplementary Information:**

The online version contains supplementary material available at https://doi.org/10.1186/s13011-026-00743-4.

## Introduction

The opioid overdose crisis continues to overwhelm communities across the United States, with Kentucky among the states hardest hit by opioid-related overdose deaths [1, 2]. In response, medications for opioid use disorder (MOUD), especially buprenorphine and methadone, have become the gold standard for treatment, significantly reducing the risk of overdose and death compared to non-medication-based approaches [3]. Yet, even with the clinical effectiveness of these medications, MOUD programs often face persistent challenges related to patient engagement, fragmented services, and difficulty retaining individuals in care [4].

To address these barriers, some treatment programs have introduced care navigators into their service models, which have seen success in other medical venues [5, 6]. Care navigators are staff who provide critical logistical and systems-level support for patients with complex needs, including health-related social needs. Their work often includes helping patients navigate transportation, housing instability, insurance issues, and referrals to physical, mental, and dental health services [7–9]. Despite their growing presence in medical settings, little is known about how these roles have fared in relation to MOUD, particularly as a method to increase retention in care, though studies implementing care navigators in hospital and detention settings found that individuals who worked with navigators were more likely to initiate MOUD treatment [8, 10–12]. Further, little research exists on how they are perceived and integrated by treatment center staff responsible for delivering day-to-day care.

For the HEALing Communities Study (HCS), the HCS-Kentucky (HCS-KY) team developed *HEALing Transitions*, a novel care navigation program designed to enhance linkage and retention in MOUD treatment. The program embedded clinical care navigators, who were typically licensed nurses and social workers, into MOUD programs through a partnership with Bluegrass Care Navigators (BCN), a third-party vendor contracted by HCS-KY. BCN managed the hiring and supervision of care navigators, providing standard organizational training, while the HCS-KY offered over 60 h of specialized training focused on MOUD, mitigating adverse health outcomes, and patient engagement [9].

The HCS-KY Implementation Facilitators, specialized staff who were trained in organizational change methods, supported treatment centers by introducing care navigators and conducting ongoing check-ins to assess relationships between care navigators, agency staff, and patients. Distinct from Medicaid-recognized Targeted Case Managers (TCMs), who typically provide high-intensity clinical services, care navigators offered more flexible, patient-centered relationship-based support to promote treatment continuity and improve patient retention [13, 14], with the frequency of visits based on a formal assessment of risk of treatment discontinuation. Care navigators primarily focused their time on addressing barriers to continued treatment, such as transportation, housing, and other basic needs, by connecting patients to community resources. They also frequently facilitated referrals to additional medical or social services. Care navigators were hired into full-time positions through BCN. While their positions were full-time, their time was often distributed part-time across two or three separate agencies; the only exception was when an MOUD program had a large patient census (over 500), which constituted a full-time assignment at a single agency. Care navigators also had access to barrier relief funds so that when community resources did not meet patient needs, funding for transportation, emergency housing, or other essentials could be provided by the HCS.

This qualitative study explores how staff within MOUD treatment centers viewed the implementation of care navigators, highlighting their perceived strengths, limitations, and fit within existing workflows. By capturing insights from frontline staff, the study sheds light on the real-world dynamics of integrating care navigators hired and trained by an outside organization into complex clinical environments. These findings aim to inform future implementation strategies that support successful integration of care navigators into MOUD treatment settings, ultimately contributing to improved care coordination and patient retention.

## Methods

### Study design and context

This qualitative study was nested within the larger, multi-site, parallel-group, cluster-randomized waitlist-controlled HCS, which tested the impact of the *Communities That HEAL* (CTH) intervention, a community-engaged strategy designed to reduce opioid overdose deaths through expanded implementation of evidence-based practices, including MOUD [15]. Community coalitions participating in HCS-selected strategies to expand MOUD capacity, improve linkage to treatment, and/or enhance MOUD retention [3, 16]. The intervention period spanned January 1, 2020, through June 30, 2022. The present qualitative study was designed to explore staff perceptions of the integration and implementation of care navigators, a commonly selected retention strategy among MOUD agencies. All study procedures were approved by a single Institutional Review Board (Advarra Pro00038088), including those specific to this qualitative data collection.

Care navigation was implemented using a largely standardized model across participating agencies. Care navigators were employed and supervised by BCN, received consistent baseline training from both BCN and HCS faculty, and were supported through ongoing implementation facilitation. In most cases, care navigators supported more than one agency. Variation across sites primarily reflected differences in allocated navigator time (e.g., 0.25–1.0 full-time equivalent) as well as agency-level onboarding and integration practices. Caseloads, agency census, and staff-to-patient ratios also varied across sites and were not systematically collected as part of this study.

### Recruitment and data collection

Between January and March 2023 (approximately 6–8 months following the conclusion of the intervention period), we conducted semi-structured interviews with staff from MOUD partner agencies that had implemented retention strategies during the first wave of the HCS, which included eight KY counties. These strategies included patient transportation support and placement of peer support specialists and/or care navigators (with barrier relief resources) in treatment centers. Additional File 1 provides an abridged version of the interview guide related to the MOUD care navigator strategy; the interview questions were designed to reflect key elements of the Reach, Effectiveness, Adoption, Implementation, and Maintenance (RE-AIM) framework and the Practical, Robust Implementation and Sustainability Model (PRISM) [17–20]. We used purposive sampling to ensure a range of agency characteristics, including representation from both urban and rural counties and from agencies that were affiliated with HCS coalitions in each county as well as those that were not. Thirty-three MOUD agencies were contacted by email or phone to participate. Small group interviews were prioritized, though individual interviews were conducted if only one staff member was available. In total, 23 interviews were conducted with agency partners that implemented at least one MOUD retention strategy, comprising 11 small-group interviews and 12 individual interviews, for a total of 38 participants.

This manuscript focuses on data from 29 participants employed by 16 methadone- and/or buprenorphine-providing treatment agencies that implemented care navigation to help retain their clients on MOUD; the remaining nine participants were affiliated with agencies that implemented other retention strategies but did not include care navigation and were therefore excluded from this analysis. Nine of the agencies were office-based opioid treatment (OBOT) centers that primarily provided buprenorphine products, while the remaining seven were opioid treatment programs (OTPs) that offered methadone. All participants provided verbal informed consent and were offered a $50 Amazon gift card; no participants declined or were prohibited from receiving compensation. Interviews were conducted via videoconference or telephone, audio-recorded, and professionally transcribed. Interviews were conducted by trained qualitative researchers and implementation staff affiliated with the HCS; some interviewers had pre-existing professional interactions with participating agencies. Additional details are provided in the COREQ checklist (Additional File 3).

Across sites, interviews included between one and three participants per agency. Although participant roles were not systematically recorded, interviewees represented a range of positions within treatment organizations, including clinical staff, administrative leadership, case managers, and peer support personnel. In small-group interviews, participants often held different roles within the same organization, allowing for multiple perspectives on care navigation implementation. Demographics were compiled using a combination of the Partner Organization Qualitative Demographic Classification Sheet, study records, and notes from Kentucky-based study personnel. Table 1 provides a summary of interviewee demographic characteristics. Barrier relief data were collected and recorded in Qualtrics from 2020 to 2022 and REDCap project version 14.4.1 in 2023 by staff from Bluegrass Care Navigators.

**Table 1:** Demographic characteristics of interview participants (*n* = 29)

Age	% (*N*)
18–34 years	27.6% (8)
35–49 years	48.3% (14)
50–64 years	20.7% (6)
65–74 years	3.5% (1)
Sex	
Female	93.1% (27)
Male	6.9% (2)
Ethnicity	
Hispanic/Latino/a/e	6.9% (2)
Non-Hispanic	93.1% (27)
Race	
White	93.1% (27)
Black	6.9% (2)
Educational attainment	
Some college, no degree	6.9% (2)
Associate degree	10.3% (3)
Bachelor’s degree	24.1% (7)
Master’s degree	55.2% (16)
Professional degree (e.g., MD, DDS)	3.5% (1)
Facility type	
Opioid treatment program (OTP)	17.2% (5)
Office-based MOUD treatment program	82.8% (24)

Note: Percentages may not sum to 100% due to rounding error

### Data analysis

We employed a two-phase coding and thematic analysis process [21, 22] integrating both deductive and inductive approaches. In the initial phase, a team of 11 trained research staff deductively coded all passages related to care navigator roles and experiences. The deductive codebook is described in Jadovich et al. (In Press) [23]. The RE-AIM and PRISM frameworks were used as interpretive lenses to guide thematic organization and interpretation of findings rather than formal coding frameworks. Initial coding was deductive, with subsequent analysis informed by these frameworks to situate findings within implementation science constructs [17–20].

For the second phase, one researcher developed an inductive codebook specifically focused on care navigator content. This codebook was piloted by two additional coders who independently applied the codes to several interview excerpts. The team regularly met to revise definitions and resolve discrepancies, refining the codebook until full consensus was reached. Subsequently, three researchers performed consensus coding of the care navigator excerpts using the finalized codebook. The team then used thematic analysis to identify key patterns related to the perceived value, integration, and challenges of the care navigator role in MOUD treatment settings. Consistent with qualitative methodology and thematic analysis, we did not quantify theme frequency or calculate proportions of respondents, as themes were not mutually exclusive and the study was designed to capture variation in perspectives rather than prevalence. Next, the three researchers individually selected passages that represented the key themes and then reached a consensus on which ones to include in the manuscript. The inductive codebook specific to this analysis is available in Additional File 2. Additional File 3 contains our consolidated criteria for reporting qualitative research (COREQ) checklist [24].

## Results

Four interrelated themes emerged from the interviews: early value recognition, impact on agency culture and team dynamics, client engagement through barrier relief, and challenges to integrating and implementing the care navigator role. While themes varied in prominence, each was represented across multiple participants and sites and often co-occurred within the same interviews. Rather than representing distinct “positive” or “negative” categories, these themes capture the complexity of how care navigators were experienced in practice. Each theme is explored below, with illustrative quotes from staff to contextualize their experiences.

### Early value recognition

Staff reported an immediate recognition of the value that care navigators could bring to their organizations. Even before the role was fully integrated, some staff perceived care navigators as capable of addressing longstanding service gaps, particularly those related to patient’s non-clinical needs. These included logistical challenges such as transportation, housing, and coordination of the wraparound services that clinical staff either did not have the time or capacity to offer.

This early enthusiasm was evident in one staff member’s reflection about why they desired care navigator implementation:

I think that it was just understanding the impact that it was going to have on our patients. I mean, it was kind of a no-brainer. Anything that’s going to help them, why wouldn’t you do it? (01152446, OTP)

Staff recognized that the care navigators were coming in with a great deal of training, experience, and knowledge. Often, the care navigators were able to assimilate into a role quickly because of this training and their knowledge of community resources. As one staff member mentioned:

[Care navigator] came at a really good time because here I am trying to train this [newly employed internal case manager] virtually and let her know what’s available in the area, and it was just really difficult. So [care navigator] came in here and just knew. She already knew what was available. She already knew who to reach out to. She had contacts at places, and it was really beneficial, especially during the time we were in. (01022768, OBOT)

Their comments encapsulated a common sentiment that care navigators were seen as essential, not auxiliary, to patient-centered care. By handling practical and immediate needs, care navigators often freed up clinical staff to focus on counseling and medical interventions while helping address patients’ basic needs (e.g., food, housing, transportation).

### Impact on agency culture and team dynamics

The addition of care navigators was described by many staff as transformative to agency culture and workplace morale. Several participants noted that seeing consistent improvements in clients’ outcomes contributed to a renewed sense of purpose and cohesion among team members. One provider commented:

I think it boosted morale because I think the teammates in the agency were actually seeing improvements within the patients. I would say during COVID and before the HEALing Communities Study came in, we had a lot of turnover and stuff. But I also feel like these ladies that came in, especially the second care navigator…like I said a while ago, they have helped with that morale within us. And whenever the patients are seeing us come together and communicate and work well together, they’re being more positive about their recovery because they see more people care. (01032376, OBOT)

Staff also appreciated the relief that navigators provided by sharing the workload, particularly in settings where resources were limited and staff were stretched thin. Staff noted this benefit of care navigators:

*Interviewee 1*: And when you don’t have people like that, I’m only one person. I can’t do it all myself. And when I had those girls [care navigator and peer support specialist] with me and helping me boost that, it was fabulous. It helped so much.

*Interviewee 2*: It took a lot of work off of our clinicians because I know [clinician] at [treatment center] spent a lot of time trying to case manage before we had a case manager. And it was very difficult a lot of times for the clinicians to wear both hats.

*Interviewee 1*: Well, like I mentioned before, it was able to delineate, it was able to let the clinicians focus on clinical work and not take up so much of their time doing case management work. (01032376, OBOT)

This care navigator support often improved workflow efficiency and fostered a sense of shared responsibility for patient success, consistent with PRISM’s focus on team integration and organizational fit. In agencies where care navigators were effectively integrated into care teams, the role was widely embraced as a complement to clinical services, contributing to a more collaborative treatment environment. This integration was facilitated by clear role definition, active communication between care navigators and clinical staff, intentional onboarding processes that aligned care navigator activities with existing workflows, and inclusion of care navigators in team meetings and care coordination processes.

### Client engagement through barrier relief

Staff overwhelmingly described the care navigators’ ability to provide direct barrier relief as their most valuable contribution. Care navigators were frequently credited with addressing patients’ social determinants of health including housing stability, transportation, and other basic needs, a key mechanism supporting reach and sustained engagement in RE-AIM. When community resources were not available to meet clients’ needs, care navigators had access to barrier relief funds through the HCS. Across agencies, care navigators distributed approximately $238,000 in barrier relief funds to 550 individuals, most commonly for rent, emergency housing, transportation, and cell phones (See Table 2). These resources illustrate the scope and types of needs addressed through care navigation.

**Table 2:** Barrier relief funds distributed by care navigators

Barrier Relief	Unique Clients	Total Cost	Per Client Cost
Transportation^1^	72	$6,529	$91
Cell Phones^2^	109	$10,800	$99
Rent^3^	100	$86,301	$863
Emergency Housing^4^	87	$58,302	$670
MOUD Other^5^	182	$75,996	$418
Totals	550	$237,927	$433

^1^Transportation – Bus passes, fuel cards, and ride services; not limited to MOUD-related appointments

^2^Cell phones – Mobile phone purchase and short-term service (up to two months)

^3^Rent – Partial or full rent assistance paid directly to lessor (up to $1,200)

^4^Emergency Housing – Short-term housing support (up to 14 days; maximum $1,200)

^5^MOUD Other – Includes utilities, identification documents, vehicle repair, prescriptions, and other healthcare-related costs (excluding clothing)

Detailed definitions and eligibility criteria for barrier relief categories are provided in Additional File 4

These community- and HCS-supplied barrier relief funds came up frequently in interviews. One participant emphasized:

That was wonderful because she didn’t only provide Narcan or anything like that. She was helping from—with other resources, and a lot of people looked forward to talking with her because we had a lot of people that were in need of housing, just helped with the utilities. She helped with a little bit of everything, and the people really connected with her. She was a very easy person to talk with, and they all loved her. (01082401, OBOT)

Another staff member from a different agency reinforced this point:

It’s kind of like [patients] were getting that “reward” when they finally got here. I think what would benefit it more is being able to advertise that we offer things like that for certain counties out in the public, because I’ve seen once people were able to meet [care navigator] for the first time and kind of see, “Oh, this isn’t just me applying for this like I have in the past and it not working and being denied,” whenever the barriers they had before, but actually seeing the results pretty quickly. Because usually she—with those patients—she would work with them as soon as they came in. That’s where we’re obviously, they’re coming from the street, they’re coming out of rehabs, things like that, and there’s a lot of just up in the air, first couple months of recovery.

That’s where the retention services were needed the most. We’re more likely to have four or five patients that just started a month ago leave treatment, than someone that’s been here a year. Just targeting those new intakes and the people that were maybe the first time in treatment or whatever, I think we’ve seen a lot of people stay in treatment because they were able to meet with her and get those services as soon as they got here. Whether that be on the HEAL grant for their treatment coverage or coming here and signing up for the busing, whatever, it’s just kind of like we hit the problem head on at the door, and I think that helped a lot. (01092415, OTP)

These supports were particularly critical for patients early in treatment and recovery, whose ability to attend appointments and adhere to treatment plans was often contingent on resolving immediate basic physiologic, safety, and social needs fundamental for survival. One exchange highlighted this need:

*Interviewer:* As soon as possible, because that is the most critical point, is that once they access your facility and your services, it’s trying to retain them at that point. It takes—.

*Interviewee:* Right, because that’s already the big step, and I feel like when they’re coming in, into recovery, we’re looking for any reason to hit the door running and prove that it won’t work. So they’re looking for any kind of barrier, “Oh, I can’t do it because of this.” Well, let’s get rid of that barrier, so what’s your next excuse? It’s kind of just minimizing what they can use an excuse to return to use. (01092415, OTP)

The cumulative effect of barrier relief translated into measurable improvements in patient retention, corresponding to effectiveness and maintenance domains of RE-AIM. Several staff attributed higher retention rates to the presence of care navigators and their accompanying ability to locate barrier relief funds. As one explained:

She had access to funds and community resources that we didn’t, and still don’t, things that were specific to Bluegrass Care. So some patients that were really struggling, she was able to address things that were maybe not the first thing you’d think about, but that were barriers to the patient themselves. Probably the best example of that is a patient we had who was really struggling, and part of her mental health concerns that was keeping repetitive ups and downs going was her teeth, and her insurance wouldn’t cover to have them repaired. The self-esteem issue was a big concern for her, and part of what would drive her back to, not necessarily opiate use, but some sort of substance use, was able to get her into a program where they paid to have her teeth fixed, which helped with her getting a job, and sustaining employment, and then sustaining remission. (01072810, OBOT)

These observations suggest that by addressing unmet health and material needs, care navigators helped create conditions more conducive to sustained engagement in MOUD treatment.

### Challenges to integrating and implementing the care navigator role

Despite the overall positive assessment, several staff members noted challenges in effectively implementing and integrating the care navigator role. In some cases, the care navigator lacked personal lived experience with substance use or the structure of the opioid treatment programs (OTPs), which OTP staff believed limited their ability to build rapport with patients; they couldn’t offer “the relatability perspective that the clients really appreciate” (01072807, OTP). Other staff identified more operational barriers, including delays in placement and scheduling limitations. One respondent noted that care navigators were minimally present on site and, in one instance, difficult to reach by phone:

*Interviewer*: How successful was the [care navigator] in reaching those people that you had hoped that they would reach?

*Interviewee*: I mean, I would say minimally successful probably, because again, she wasn’t here on site and so often—well, I mean, she was, but for a very short amount of time, two, three hours at most. And so a lot of times the clients that would need her were not available, and they’re not great about answering their phones. (01092412, OTP)

Referring to early mismatches between agency needs and navigator availability, another staff member noted the logistical challenges of shared assignments:

“I’m at [other treatment center] that day,” or, “[other treatment center] needs me.” Okay, I understand that. I get that you’re working two sites, but you were not so busy that you couldn’t help with ours. She had, at best seven people at a time on her caseload with us, which we had been told it would be a little bit more. We stopped referring to her because the people that she even had, I ended up doing the work. I ended up doing the work. So not only did her inaction of not being here and us getting phone calls from clients about not being able to reach her about things that had been promised cause additional work, but it caused additional work to me because these things that she had promised, they had to be completed. So guess who did it? It was the counselors. Very disappointed. Very disappointed in her. (01083214, OTP)

Strict client eligibility protocols and paperwork requirements also posed barriers to effective care navigation. When a client’s needs could not be met by existing community resources or were outside standard support categories, the HCS was able to approve and fulfill non-standard requests for barrier relief (see Table 2). One participant shared:

I do think on some resources, they were helpful. I wish all resources were available to everyone, but because the resources were divided and some of them had different access, that did make it beneficial to have both. But to her point, I felt like there was more almost like red tape you had to get through with the Care Navigator than there was with the peer support. I don’t know if the paperwork should have been the same or if it was different, but the perception of getting someone into those services seems like there was more hoops to jump through. (01072807, OTP)

These constraints sometimes created misalignment between the role’s intended flexibility and its practical execution with patients. One staff member observed,

I think fundamentally, [care navigator] policies are rigid…and therein lies the problem, because you can’t be with this patient base. They don’t show up on the day that they’re here and things like that. But the intake process and the paperwork, sometimes you sit down and help the patient and then figure out the rest. I thought that was a major difference in how the agencies rolled out their people to us. (01072807, OTP)

Taken together, these challenges reflect variability in how the role was implemented across sites, consistent with differences in implementation and organizational context described in RE-AIM and PRISM. While many care navigators were viewed as flexible and client-centered, others were described as struggling to meet expectations, particularly regarding role clarity, supervision, and alignment with agency practices.

## Discussion

Guided by RE-AIM and PRISM as interpretive frameworks, this study reflects the diverse perspectives of MOUD treatment center staff on the integration and impact of care navigators within their agencies. Participants highlighted both the promise and complexity of the role, identifying its key strengths in client engagement and operational support, as well as challenges related to role clarity and implementation logistics.

Care navigators were widely perceived as filling critical implementation gaps in MOUD programs, particularly in enhancing the Reach and Implementation dimensions, through retention of clients, of the RE-AIM framework (Glasgow et al., 2011). Staff consistently reported that care navigators helped retain patients by addressing practical barriers to care, such as transportation, housing, and medical access. These findings reinforce earlier research showing that individuals performing non-clinical service can effectively improve treatment retention [13].

Aligning with the PRISM framework, care navigators were viewed as instrumental to organizational climate and team functioning. Staff members noted morale improvements and reduced burnout attributed to care navigator support, aligning with evidence that new roles can improve workplace culture and team cohesion [25]. Team members described feeling more supported and resilient with improved organizational resources [26].

Nevertheless, persistent challenges emerged affecting navigator integration. Staff frequently expressed confusion about care navigators’ roles, especially in relation to existing positions like targeted case managers and peer support specialists. Scheduling inconsistencies and unclear job duties created overlaps and occasional friction. These problems underscore the importance of readiness for change and cultural fit; in several sites, care navigator policies or professional demeanor differed from the MOUD center expectations, leading to misalignment and strained expectations.

These difficulties echo broader implementation science findings. Adeniran et al. (2023) highlights that ambiguous job responsibilities and poor scheduling systems are common when new roles are introduced rapidly without sufficient planning and delineation. Greater attention to clearly defining care navigators’ focus on short-term, logistical support, as distinct from TCMs’ clinical, Medicaid-billable case management, may reduce role tension. In this context, care navigators can extend support to individuals not eligible for Medicaid-funded services or address needs not covered by billable care, provided that roles are well coordinated and clearly delineated to avoid duplication [27].

These findings offer several practice and policy implications. First, the sustainable maintenance of care navigator positions requires stable and flexible funding for agencies. The HCS provided the initial funding for these roles, underscoring the necessity of dedicated financial support for other organizations to successfully implement or continue this service. This might include grants, value-based contracting, or public-private partnerships [28]. Second, training and onboarding protocols should explicitly define role boundaries, supervisory structures, and strategies to avoid overlapping responsibilities with existing clinical staff. Clear messaging can support buy-in and collaboration. Finally, investing in staff orientation and ongoing implementation facilitation is essential for success. This sustained support, including regular check-ins by implementation facilitators with clinic staff and joint troubleshooting, can help align organizational culture and foster the integration of care navigators as effective team members. Given the variability in regulatory oversight and patient needs across different settings, this ongoing adaptability is critical for successful implementation.

A number of limitations should be noted. This study draws exclusively on staff perspectives and does not include the voices of MOUD patients or care navigators themselves, whose experiences may differ in meaningful ways. Data were collected from treatment centers in Kentucky, which may limit the transferability of findings to other geographic or policy contexts. All insights stem from semi-structured interviews; no observational, clinical, or retention outcome data were presented. In addition, participant roles were not systematically recorded, limiting our ability to assess how perspectives may have differed across clinical, administrative, and support staff positions. Furthermore, the care navigator role varied across settings and was still evolving during the study period, potentially influencing how participants understood and described its impact. We did not systematically collect detailed data on staffing structures, care navigator caseloads, or agency-level characteristics (e.g., patient census or staff-to-patient ratios), limiting our ability to assess whether observed experiences were associated with specific organizational contexts. As with all qualitative work, there is potential for social desirability bias, particularly given the preexisting relationships between some interviewers and agency staff. These limitations suggest the need for broader, multi-perspective, and mixed-method studies to assess the role and effectiveness of care navigators more comprehensively.

## Conclusion

Care navigators were consistently described by MOUD treatment staff as addressing critical service gaps, particularly by enhancing patient engagement and resolving everyday barriers to care. These findings support the growing recognition that integrating care navigation staff into addiction treatment teams can improve not only patient support but also staff morale and organizational cohesion. Clearer delineation between care navigators, targeted case managers, and peer support specialists is essential for reducing role confusion and maximizing team function. Taken together, the results suggest a need to reevaluate MOUD staffing models to better support holistic, person-centered care that extends beyond the clinical setting. Future research should include patient, navigator, and implementation facilitator perspectives and examine the impact of care navigation on treatment retention and recovery outcomes. Cross-jurisdictional studies may also help clarify how implementation strategies and policy environments shape the effectiveness and sustainability of these roles.

## Supplementary Information

Below is the link to the electronic supplementary material.

Supplementary Material 1

Supplementary Material 2

Supplementary Material 3

Supplementary Material 4

## Data Availability

The qualitative interview data generated and analyzed during the current study are not publicly available due to confidentiality protections and the potential for participant identification; however, data may be made available from the corresponding author upon reasonable request and with appropriate approvals.

## Abbreviations

BCN: Bluegrass Care Navigators
CDC: Centers for Disease Control and Prevention
COREQ: Consolidated Criteria for Reporting Qualitative Research
CTH: Communities That HEAL
HCS: HEALing Communities Study
HCS-KY: HEALing Communities Study in Kentucky
HEAL: Helping to End Addiction Long-term^®^
IRB: Institutional Review Board
MOUD: Medication for opioid use disorder
NIH: National Institutes of Health
OBOT: Office-based opioid treatment
OTP: Opioid treatment program
PRISM: Practical, Robust Implementation and Sustainability Model
RE-AIM: Reach, Effectiveness, Adoption, Implementation, Maintenance
SAMHSA: Substance Abuse and Mental Health Services Administration
SAS: Statistical Analysis Software
TCM: Targeted Case Manager
