# Supplementary material for: Staff perceptions of care navigators in medication for opioid use disorder treatment settings
Source: Subst Abuse Treat Prev Policy. 2026 Jun 27;21:60. doi: 10.1186/s13011-026-00743-4 (PMC13599137; doi:10.1186/s13011-026-00743-4)
Supplement: Supplementary file 1 [file 13011_2026_743_MOESM1_ESM.docx]

**Staff Perceptions of Care Navigators in Medication for Opioid Use Disorder Treatment Settings**

**Additional File 1**

**Sustainability Partner Organization Interview Guide**

***Instructions to Interviewer:*** This guide is for Wave 1 communities ONLY. It should be tailored to address the specific EBPs implemented by the organization (i.e., skip sections not relevant to EBPs that were not implemented). Prior to the interview, you should discuss with your team lead about which sections are relevant, based on the sampling plan for your site. You should also review implementation data (e.g., ORCCAT, Reach) related to the partner organization to understand what has been implemented. To obtain information that is as complete as possible, probes should be used if a participant does not specifically address each of the sub-questions in their response to the main question.

Before beginning the interview, please record the interview date and start time below. Please record the interview end time at completion of the interview.

**Interview Date: ____________________________**

**Interview Start Time: __________________**

***Brief introduction to be read to interviewee:***

First, THANK YOU for agreeing to participate in this interview.

As background, these interviews are being conducted with various organizations that participated in implementing evidence-based practices, or EBPs, as part of the [*use site-specific nomenclature such as: HEALing Communities Study (HCS)/Communities That HEAL (CTH) intervention, HEAL grant*]. We want to learn about your experiences with implementing these EBPs within your organization during the time of the study and also what has happened in the months since the end of the intervention period.

# Menu 2: Medication for Opioid Use Disorder (MOUD)

***Instructions to Interviewer: Ask the following if (1) organization implemented one or more MOUD strategies/interventions and/or expanded one or more MOUD strategies/interventions during Wave 1 as part of the HCS/CTH and (2) the organization has been selected for sampling about MOUD implementation. An organization may have implemented multiple Menu 2 strategies, in which case it is important to try to delineate similarities or differences between the Menu 2 strategies, such as whether barriers and facilitators were similar for the multiple strategies; repeat questions for the various Menu 2 strategies if necessary to understand similarities/differences. If the organization is not an MOUD partner or has not been sampled for the MOUD section, skip this section, and move to the Final Question.***

1. Let’s discuss your organization’s experience with implementing strategies related to medication for opioid use disorder, or MOUD, as part of the [*use site-specific nomenclature such as: HEALing Communities Study (HCS)/Communities That HEAL (CTH) intervention, HEAL grant*]. These MOUD-related strategies may have included improving access to MOUD, improving linkage to MOUD, and/or improving retention on MOUD. My understanding is that your organization implemented [… insert information from records/ORCCAT about what strategies from Menu 2 were implemented]. Is that accurate?
   - **Probes**
     - What did[this/these] MOUD-related [*strategy/strategies/or other site-specific nomenclature about the strategy/strategies]* look like for your organization during HCS?
     - In what ways did implementation involve coordination with other organizations?
     - How did these MOUD strategies compare to what your organization was doing prior to HCS?
     - Who were you trying to reach with these MOUD strategies? How successful was your organization in reaching them?

2. What were the biggest factors that helped your organization implement these MOUD-related strategies during the HEALing Communities Study, or HCS?
   - **Probes**
     - In what ways did [*use site-specific nomenclature such as: HEALing Communities Study (HCS)/Communities That HEAL (CTH) intervention, HEAL grant*] help your organization with implementing these MOUD-related strategies?
     - ***Ask additional probes*** ***for facilitators relevant to your state’s research site,*** *such as coalition support, the coalition’s policy influence, learning collaboratives, monetary resources, help with staffing, help designing interventions, provision of TA, help with monitoring, etc.*

3. What were the biggest barriers or challenges that your organization faced during its implementation of these MOUD-related strategies during HCS?
   - **Probes**
     - ***Ask additional probes*** ***for barriers relevant to your state’s research site,*** *such as incompatibility with organizational culture/mission, stigma, competing demands, lack of funding, lack of support, COVID pandemic*
     - In what ways, if any, did [*use site-specific nomenclature such as: HEALing Communities Study (HCS)/Communities That HEAL (CTH) intervention, HEAL grant*] help to address barriers?
     - In what ways, if any, did [*use site-specific nomenclature such as: HEALing Communities Study (HCS)/Communities That HEAL (CTH) intervention, HEAL grant*] hinder your organization’s implementation of these MOUD-related strategies?

▪ ***Probe*** *if changing demands by the study was a challenge, such as changing data requirements and reporting, or implementation planning*

4. How would you describe the overall impact of implementing these MOUD-related strategies during HCS on your organization?

5. How would you describe the overall impact of these MOUD-related strategies during HCS on [*your patients/your clients/the people that it serves*]?

6. The intervention period for [*use site-specific nomenclature such as: HEALing Communities*

*Study (HCS)/Communities That HEAL (CTH) intervention, HEAL grant*] ended on June 30^th^ of 2022. To what extent has your organization continued these HCS MOUD-related strategies since June 30^th^, 2022?

- ***If some strategies have been sustained but others have not, or it is unclear what has been sustained, ask for clarification:*** Which MOUD-related strategies have continued, and which have ended?

***7A. If any or all of the HCS MOUD strategies have continued (even if adaptations have been made), ask:***

7A1. What changes, if any, has your organization made to these MOUD-related strategies since June 30^th^? What factors led to those changes?

7A2. What has helped your organization to sustain [this/these] MOUD-related

[*strategy/strategies/or other site-specific nomenclature about the strategy/strategies that have been sustained*]? o **Probes**

- What factors internal to your organization have helped to sustain [this/these] MOUD-related [*strategy/strategies/or other site-specific nomenclature about the strategy/strategies that have been sustained*]?
- What factors outside your organization have supported your ongoing implementation of [this/these] MOUD-related [*strategy/strategies/or other site-specific nomenclature about the strategy/strategies that have been sustained*]?

7A3. What, if anything, has made it difficult for your organization to sustain [this/these] MOUD-related [*strategy/strategies/or other site-specific nomenclature*]? o **Probe**

- ***If any barriers are identified, ask:*** To what extent have those barriers been overcome? What has helped your organization to overcome those barriers?

7A4. How likely do you think it is that your organization will sustain [this/these] MOUD related [*strategy/strategies/or other site-specific nomenclature*] over the long term, such as over the next 1-2 years?

7A5. What is likely to help or hinder your organization’s ability to sustain [this/these] MOUD-related [*strategy/strategies/or other site-specific nomenclature*] over the next 1-2 years?

***7B. If any but not all HCS MOUD strategies have ended, continue by asking:***

7B1. Can you tell me more about the MOUD-related [*strategy/strategies/or other site-specific nomenclature*] that has/have ended?

7B2. What made it difficult to continue [this/these] MOUD-related [*strategy/strategies/or other site-specific nomenclature*]?

• **Probes**

- What factors inside and/or outside your organization made it difficult to continue [this/these] MOUD-related [*strategy/strategies/or other site-specific nomenclature*]?
- ***Probe*** *about state or local policies, lack of funding, stigma, lack of community support.*

***7C. If all HCS MOUD strategies by the organization have ended completely, ask:***

7C1. Can you tell me about what made your organization choose to end [this/these] MOUD-related [*strategy/strategies/or other site-specific nomenclature*]?

7C2. What made it difficult to continue [this/these] MOUD-related [*strategy/strategies/or other site-specific nomenclature*]? o **Probes**

- What factors inside and/or outside your organization made it difficult to continue [this/these] MOUD-related [*strategy/strategies/or other site-specific nomenclature*]?
- ***Probe*** *about state or local policies, lack of funding, stigma, lack of community support.*

**Final Question [all organizations]**

Looking back at everything your organization implemented because of HCS, what were the impacts on the community?
