# Supplementary material for: Staff perceptions of care navigators in medication for opioid use disorder treatment settings
Source: Subst Abuse Treat Prev Policy. 2026 Jun 27;21:60. doi: 10.1186/s13011-026-00743-4 (PMC13599137; doi:10.1186/s13011-026-00743-4)
Supplement: Supplementary file 2 [file 13011_2026_743_MOESM2_ESM.docx]

**Staff Perceptions of Care Navigators in Medication for Opioid Use Disorder Treatment Settings**

**Additional File 2**

**Inductive Codebook: Care Navigators**

**Overview and Process Summary:**

This codebook was created for the HCS to systematically code interviews with organizational partners who implemented MOUD-related strategies during Wave 1 (January 2020 – June 2022). This coding structure includes parent, child, and grandchild codes to capture different study dimensions.

Interviews were conducted approximately 6–8 months after the end of the intervention period to gather partner perspectives on the implementation and sustainability of EBPs. In Kentucky, these interviews were conducted by Implementation Facilitators, many of whom assisted partners with evidence-based practices (EBPs) during the intervention.

This codebook focuses on interview passages that reference the care navigators from Bluegrass Care Navigators who were deployed within MOUD agencies. Additional child codes were developed deductively by HCS-KY researchers, who initially reviewed all partner interview passages coded in the Kentucky (KY) data as part of a cross-site process. After this initial review, researchers drafted child code definitions along with inclusion and exclusion criteria to code passages about the implementation of care navigators as an intervention to increase retention on MOUD.

A small team of coders who had previously conducted Partner Sustainability interviews and/or participated in cross-site coding then applied the preliminary child codes to a sample of five pages of care navigator excerpts. This step ensured consensus in coding application, identified areas for refinement, and evaluated whether additional child codes emerged from the data.

| **Adoption** | Definition: Statements about why an agency desired a care navigator before implementation took place.  Inclusion criteria:   - Include descriptions of why the agency knew a care navigator would be useful for the agency.   Exclusion criteria:   - Exclude statements that **infer** a desire for a care navigator prior to implementation. |
| --- | --- |
| **Barrier Relief Community Offerings** | Definition: Statements about how care navigators provide connections to community resources for barrier relief **without** the use of barrier relief funds.  Inclusion criteria:   - Include descriptions of care navigators using community resources to assist a patient with barriers to care (e.g., external grants). - Include descriptions of care navigators assisting clients in finding employment without the use of barrier relief funds. - Include descriptions of how scheduling care navigators assisted with HUD/housing without the use of barrier relief funds. - Include descriptions of care navigators using community resources similar to what agency staff used in the past, without barrier relief funds.   Exclusion criteria:   - If statements about barrier relief do not explicitly state the use of external funding and likely have a cost, code to “Barrier Relief Funds.” |
| **Barrier Relief Funds** | Definition: Statements about how the care navigators provided **fiscal** support to patients in need using HCS funding.  Inclusion criteria:   - Include descriptions of how care navigators assisted with transportation with barrier relief funds. - Include descriptions of how care navigators assisted with fees (driver’s license, birth certificate) with the use of barrier relief funds. - Include descriptions of how care navigators assisted with patient bills. - Include descriptions of how care navigators assisted with housing with the use of barrier relief funds.   Exclusion criteria:   - Statements about barrier relief that are known to be from community funds should be coded, “Barrier Relief Community Offerings.” |
| **Client Characteristics** | Definition: Statements about who care navigators served.  Inclusion criteria:   - Include discussions of how the agencies chose patients for care navigators focus (e.g., patients at risk of dropping out, new patients, patients with co-occurring mental health issues, patients who have returned to use). - Include discussions of the benefits of reaching certain populations due to care navigator placements (patients with transportation issues, patients with financial barriers, patients unhoused).   Exclusion criteria:   - No specific exclusions. |
| **External Policies** | Definition: Descriptions of policies outside the control of agencies and HCS/BCN that affected care navigator’s work.  Inclusion criteria:   - Include statements about how HIPAA regulations influenced the ability to or method of care navigator’s work. - Include statements about how telemedicine regulations influenced the ability to or method of care navigator’s work. - Include statements about how TCM regulations influenced the ability to or method of care navigators work (e.g., TCMs not allowed in residential facilities). - Include statements about care navigators that influence care navigator’s work.   Exclusion criteria:   - No specific exclusions. |
| **Family of Clients** | Definition: Statements about how care navigators worked with family and friends of clients to create a supportive environment for treatment.  Inclusion criteria:   - Include descriptions of care navigators engaging with client/patient in rebuilding relationships with their family and friends; therefore, creating a support system   Exclusion criteria:   - No specific exclusions. |
| **Human Resources** | Definition: Statements about how the agency’s organizational structure/staffing or care navigators staffing affected the care navigator’s services.  Inclusion criteria:   - Include descriptions of how scheduling care navigators met agency needs (e.g., being available to provide services to clients/patients due to the care navigator’s work schedule). - Include statements about the management structure of the care navigators facilitated the ability to provide retention of patients. - Include statements about turnover of care navigators. - Include statements about the existence of care navigators only because of HCS funds. - Include statements about delays in placement of care navigators due to delays in BCN/HCS hiring of care navigators. - Include statements about the benefits of having additional staff available to meet with patients (e.g., expanding access to staff). - Include statements about care navigators not having to abide by billable hours. - Include statements about the process of referrals to care navigators.   Exclusion criteria:   - No specific exclusions. |
| **OEND Offerings-Agency** | Definition: Statements about how the care navigators provided OEND services **within** the MOUD treatment center.  Inclusion criteria:   - Include descriptions of how care navigators assisted with providing OEND within the treatment center.   Exclusion criteria:   - Exclude statements about how care navigators assisted with OEND in the community. Code these statements to OEND Offerings—Community. |
| **OEND Offerings--Community** | Definition: Statements about how care navigators engaged the community with OEND.  Inclusion criteria:   - Include descriptions of how care navigators provided OEND *in the community*. - Include descriptions of how care navigators helped to generally reduce opioid overdoses.   Exclusion criteria:   - No specific exclusions. |
| **Patient Engagement/Rapport Building** | Definition: Statements about how care navigators were able to build connections and trusting relationships with clients/patients in individual or group settings.  Inclusion criteria:   - Include descriptions focused on the ability of care navigators to show empathy to patients. - Include statements about lived experience or professional degrees being valuable to patient engagement. - Include statements about how care navigators deliberately attempted to build rapport (e.g., sitting in the lobby, initiating conversations, being consistent, increasing morale, passing out business cards). - Include statements about how care navigators created opportunities to work with clients individually or in groups. - Include statements of patient access to care navigators in non-traditional environments (e.g., medical offices, administrative offices, remotely). - Include statements about how the personalities of care navigators fit the needs of the patients.   Exclusion criteria:   - No specific exclusions. |
| **Stories** | This code refers to passages in the interview that are anecdotal narratives used to illustrate a point or an experience.  Notes:   - These passages may be larger chunks of the transcript. The consequences of broadly applying this code are minimal.   The Stories code should always be double coded with any other appropriate code. |
| **Care Navigator Characteristics** | Definition: Statements about the care navigator characteristics effect on integration within the agency: knowledge, training, attitude, professionalism, reliability, character.  Inclusion criteria:   - Include descriptions about how well care navigator’s professional development or training met the needs of patients and the agency. - Include statements about how the personalities of care navigators fit the needs of the agency staff or the treatment center broadly.   Exclusion criteria:   - No specific exclusions. |
