# Supplementary material for: Staff perceptions of care navigators in medication for opioid use disorder treatment settings
Source: Subst Abuse Treat Prev Policy. 2026 Jun 27;21:60. doi: 10.1186/s13011-026-00743-4 (PMC13599137; doi:10.1186/s13011-026-00743-4)
Supplement: Supplementary file 3 [file 13011_2026_743_MOESM3_ESM.docx]

**Staff Perceptions of Care Navigators in Medication for Opioid Use Disorder Treatment Settings**

**Additional File 3**

**Consolidated criteria for reporting qualitative research (****COREQ) checklist**

**Domain 1: Research Team and Reflexivity**

***Personal Characteristics***

**1. Which author/s conducted the interview or focus group?**

Authors who conducted interviews

Shaquita Andrews-Higgins

Michael Goetz

Sandra Back-Haddix

Non-Authors who conducted interviews (but not analysis for this manuscript)

Kathy Adams

Jeannie Hartman

Rachel Hoover

Latasha Jones

Hallie Mattingly

Ryan Morris

Melissa Reedy-Johnson

Courtney Rogers

**2. What were the researcher’s credentials? E.g. PhD, MD.**

The authors’ credentials are below:

Shaquita Andrews-Higgins, LPCA

Sandra Back-Haddix, MPH

Olivia A. Davis, BS

Laura Fanucchi, MD

Michael Goetz, PhD

Hannah K. Knudsen, PhD

Michelle R. Lofwall, MD

Sharon L. Walsh, PhD

Among the non-authors who conducted interviews and participated in the initial round of coding with the four state research teams, seven were master’s-level staff and one held a bachelor’s degree.

**3. What was their occupation at the time of the study?**

The researchers comprising the author team are a mix of faculty, staff, and a student. More specifically, the author team includes four faculty at the University of Kentucky: Hannah K. Knudsen, Michelle R. Lofwall, Laura Fanucci, and Sharon L. Walsh. The three staff who were employed as Implementation Facilitators/Researchers at the University of Kentucky were Shaquita Andrews-Higgins, Sandra Back-Haddix, and Michael Goetz. The writing team also includes a medical student at the University of Kentucky College of Medicine, Olivia Davis.

Seven of the eight non-authors were Implementation Facilitators, while one was an administrative assistant. All individuals in this group received the same qualitative training as the Implementation Facilitators who are authors.

**4. Was the researcher male or female?**

Researchers were comprised of both men and women.

**5. What experience or training did the researcher have?**

Interviewers received training from faculty of the four state research teams (Kentucky, Massachusetts, New York, and Ohio) on the use of the cross-site interview guide for data collection. Most interviewers had prior experience conducting qualitative interviews with a sample of coalition members also involved in the study. Following training on the cross-site codebook which was structured around the RE-AIM/PRISM framework, interviewers completed the initial coding of the transcripts. For this manuscript's additional sub-coding, the coding team, who are also authors of the paper, developed the codebook through a consensus-based process and met regularly to address coding challenges.

**6. Was a relationship established prior to study commencement?**

Initial relationships with study participants were not established prior to the start of the HEALing Communities Study (HCS) in Kentucky. However, it is a limitation that some interviewers for this manuscript's qualitative component had a pre-existing working relationship with interviewees through their involvement with scaling up evidence-based practices as part of the study.

**7. What did the participants know about the researcher? e.g. personal goals, reasons for doing the Research.**

The participants in this research were aware of the study's primary objective to reduce opioid overdose deaths through the implementation of evidence-based practices; however, the personal motivations or goals of participants were not a focus of discussion during the interviews.

**8. What characteristics were reported about the interviewer/facilitator? e.g. Bias, assumptions, reasons and interests in the research topic.**

None.

**Domain 2: Study Design**

***Theoretical framework***

**9. What methodological orientation was stated to underpin the study? e.g. grounded theory, discourse analysis, ethnography, phenomenology, content analysis.**

We employed thematic analysis to analyze the data, as described in the Methods section.

**10. How were participants selected? e.g. purposive, convenience, consecutive, snowball.**

Purposive sampling was used, as described in the Methods section.

**11. How were participants approached (e.g. face-to-face, telephone, mail, email)?**

Participants were recruited by email or phone, which is detailed in the Methods section.

**12. How many participants were in the study?**

This manuscript presents data from 29 individuals who participated in small group or individual interviews about their experiences with implementing care navigators as a strategy to increase MOUD retention.

**13. How many people refused to participate or dropped out? Reasons?**

The data for this manuscript represent a subset of agencies that implemented MOUD-related interventions. Refusals for the larger interview effort, which included agencies that had implemented overdose education and naloxone distribution and/or MOUD-related interventions, totaled 14 individuals who actively refused to participate and 39 who did not respond to repeated invitations. As this was a cross-sectional study, no participant dropout occurred.

***Setting***

**14. Where was the data collected? e.g. home, clinic, workplace.**

Interviews were conducted by interviewers working remotely. Participants were typically interviewed at their place of employment but had the flexibility to choose another location.

**15. Was anyone else present besides the participants and researchers?**

It is possible that additional individuals were present near participants during the interviews. However, due to the use of Zoom, it was not possible for the interviewer to confirm this, and the presence of others was not formally recorded as part of the data.

**16. What are the important characteristics of the sample? e.g. demographic data, date.**

Demographics are presented in Table 1.

***Data collection***

**17. Were questions, prompts, guides provided by the authors? Was it pilot tested?**

An interview guide with primary and follow-up questions was provided to interviewers. While the guide was not formally pilot tested, interviewers were required to practice using it with a colleague prior to interviews.

**18. Were repeat interviews carried out? If yes, how many?**

Repeat interviews were not carried out.

**19. Did the research use audio or visual recording to collect the data?**

All interviews were audio-recorded via the Zoom platform. The recordings were professionally transcribed and subsequently coded and analyzed.

**20. Were field notes made during and/or after the interview or focus group?**

No.

**21. What was the duration of the interviews or focus group?**

Interviews varied in length but averaged 60 minutes.

**22. Was data saturation discussed?**

Data saturation was not feasible for the scale of this data collection across the four states. Purposive sampling was used instead.

**23. Were transcripts returned to participants for comment and/or correction?**

No.

**Domain 3: Analysis and Findings**

***Data analysis***

**24. How many data coders coded the data?**

Initial deductive coding was conducted by eight coders from the four state research teams. For this manuscript, an additional round of inductive sub-coding was performed by four of the manuscript's authors (M.G., S.A.H., S.B.H., O.D.)

**25. Did authors provide a description of the coding tree?**

No. Instead, we have provided the codebook used for this manuscript, which indicates codes, child codes, and grandchild codes (see Additional File 2).

**26. Were themes identified in advance or derived from the data?**

Themes for this study were inductively derived from the data.

**27. What software, if applicable, was used to manage the data?**

Qualitative transcripts were managed using NVivo 12. The demographic data and interview completion information were entered into REDCap 14.4.1, and descriptive statistics were calculated after the data were imported into Stata 18. Barrier relief data were collected and recorded in Qualtrics from 2020 through 2022 and REDCap project version 14.4.1 in 2023 by Bluegrass Care Navigators. Statistical analyses on barrier relief data were performed using SAS, version 9.4 (SAS Institute Inc., Cary, NC).

**28. Did participants provide feedback on the findings?**

No.

***Reporting***

**29. Were participant quotations presented to illustrate the themes / findings? Was each**

**quotation identified? e.g. participant number**

Yes.

**30. Was there consistency between the data presented and the findings?**

Yes.

**31. Were major themes clearly presented in the findings?**

Yes.

**32. Is there a description of diverse cases or discussion of minor themes?**

While minor themes are referenced, a comprehensive discussion of them is not included.
