# Supplementary material for: Staff perceptions of care navigators in medication for opioid use disorder treatment settings
Source: Subst Abuse Treat Prev Policy. 2026 Jun 27;21:60. doi: 10.1186/s13011-026-00743-4 (PMC13599137; doi:10.1186/s13011-026-00743-4)
Supplement: Supplementary file 4 [file 13011_2026_743_MOESM4_ESM.docx]

**Staff Perceptions of Care Navigators in Medication for Opioid Use Disorder Treatment Settings**

**Additional File 4**

**Barrier Relief Definitions and Eligibility Criteria**

Barrier relief resources were provided to support patient engagement in medication for opioid use disorder (MOUD) treatment. The following categories outline eligibility criteria and allowable uses:

**Transportation**
Participants were required to have met at least once with a care navigator and be actively engaged in MOUD treatment. Transportation support included bus passes, fuel cards, and ride services. Use was not limited to MOUD-related appointments and could include transportation for prescription pick-up, other healthcare visits, employment, or essential needs such as grocery shopping.

**Cell Phones**
Participants were required to have met at least once with a care navigator and be enrolled in the care navigation program. Support included the purchase of a mobile phone and limited service coverage. Service was provided for up to two months, funded one month at a time.

**Rent Assistance**
Participants were required to have met at least twice with a care navigator, be actively engaged in MOUD treatment, and reside in a county participating in the intervention. Funds were paid directly to the lessor and could cover partial or full rent assistance, up to $1,200. The combined total for rent assistance, deposits, and emergency housing could not exceed $1,200 within a three-month period.

**Emergency Housing**
Participants were required to have met at least once with a care navigator, have a confirmed MOUD appointment, and have communicated with an emergency housing provider within the week prior to payment. Funds were paid directly to the housing provider and could support up to 14 days of housing within a three-month period, with a maximum of $1,200 to ensure safe or stable housing.

**Other MOUD-Related Expenses**
Participants were required to have met at least twice with a care navigator. Funding was capped at $1,500 within a three-month period. Allowable expenses included vehicle towing, repair or maintenance necessary for client transportation, jail communication fees, identification documents, utilities, prescriptions, and other healthcare-related costs. Clothing items were not eligible for reimbursement.
